# Use of Second-line Immunotherapy in Control Arms of Randomized Clinical Trials in Kidney Cancer: A Systematic Review

**DOI:** 10.1001/jamanetworkopen.2021.24728

**Published:** 2021-09-27

**Authors:** John Sharp, Ali Raza Khaki, Vinay Prasad

**Affiliations:** 1 Department of Medicine, University of California, Los Angeles (UCLA) Health, UCLA; 2 Department of Medicine, Stanford University, Palo Alto, California; 3 Department of Medicine, University of California, San Francisco; 4 Department of Epidemiology and Biostatistics, University of California, San Francisco

## Abstract

**Importance:**

Immunotherapy (anti–programmed death ligand 1 antibodies) is associated with improved survival rates in advanced kidney cell carcinoma (KCC) after progression on first-line tyrosine kinase inhibitor (TKI) treatment. It is unknown whether and to what degree patients in the control arm receive postprotocol immunotherapy in trials comparing combination immunotherapy regimens with TKI in first-line advanced KCC.

**Objective:**

To characterize the proportion of patients in the control arm who received postprotocol immunotherapy in trials comparing combination immunotherapy regimens with TKI in first-line advanced KCC.

**Evidence Review:**

A search of PubMed was conducted to identify randomized clinical trials of combination immunotherapy compared with TKI in first-line advanced KCC between January 1, 2015, and February 28, 2021. Combination immunotherapy was defined as an anti–programmed death ligand 1 agent and an additional agent. Search terms included *renal cell cancer* and *first-line* and were filtered by the type *clinical trial*. All English-language trials of combination immunotherapy compared with a TKI were included. The trials and their protocols and supplements were analyzed to determine the proportion of patients in the control arm receiving postprotocol immunotherapy.

**Findings:**

A total of 106 articles met search criteria and were screened. A total of 6 trials and 3 published updates of trial results were included in the systematic review. Of 2565 patients assigned to control arm groups, 2069 (81%) were no longer on TKI at last data cutoff. Of patients in the control arm who discontinued TKI, 932 (45%) received postprotocol immunotherapy. Of patients in the control arm receiving any type of postprotocol therapy, 66.4% received immunotherapy.

**Conclusions and Relevance:**

This systematic review found that the proportion of patients in the control arm receiving postprotocol immunotherapy is low in randomized clinical trials of first-line combination immunotherapy regimens for advanced KCC. Appropriate use of postprotocol therapy is essential to answering the question of whether a combination or sequential treatment strategy with immunotherapy is superior.

## Key Points

**Question:**

In randomized clinical trials for first-line advanced kidney cell carcinoma (KCC) comparing combination immunotherapy regimens (defined as anti–programmed death ligand 1 antibody plus an additional agent) with the tyrosine kinase inhibitor sunitinib, what is the proportion of patients in the control arm who received postprotocol immunotherapy treatment?

**Findings:**

In this systematic review examining 5478 patients enrolled in 6 trials for first-line advanced KCC comparing combination immunotherapy treatment with the sunitinib, 45.0% of the patients in the control arm who discontinued control arm treatment received postprotocol immunotherapy.

**Meaning:**

In this systematic review, use of postprotocol immunotherapy among patients in the control arm was low, which may be associated with an overestimation of the clinical benefit of the combination immunotherapy regimen.

## Teaser

This systematic review assesses the proportion of patients in the control arms of randomized clinical trials who received postprotocol immunotherapy in trials comparing combination immunotherapy regimens with tyrosine kinase inhibitor for first-line treatment of advanced kidney cell carcinoma.

## Introduction

In 2020, kidney cell carcinoma (KCC) was diagnosed in more than 70 000 adults in the US.^1^ While the 5-year overall survival (OS) rate has improved in the last several decades from nearly 50% to 75.2% in 2020,^2^ the prognosis of advanced stage disease remains stable, with a 5-year survival rate of only 13%.^3^ This is based partly on the fact that KCC is resistant to chemotherapy; consequently, a relatively limited number of treatments are known to be effective.

With the development of agents other than cytotoxic chemotherapies, options for advanced KCC treatment have increased. Sunitinib, an oral tyrosine kinase inhibitor (TKI) of vascular endothelial growth factor receptor has been a cornerstone of first-line treatment for metastatic KCC after longer progression-free survival (PFS) and OS compared with cytokine-based therapy was reported.^4,5^ Immunotherapy (anti–programmed death ligand 1 antibody treatment) has shown efficacy, with nivolumab receiving US Food and Drug Administration approval in the second-line setting after the CheckMate 025 trial reported an OS benefit.^6,7^ Recent trials^8,9,10,11,12^ in advanced KCC have sought to combine immunotherapy agents with other treatments, frequently TKIs, in the first-line setting.

When the number of effective therapies is limited, proper sequencing is important to maximize clinical outcomes for patients, such as survival and quality of life. By combining 2 effective therapeutic agents in a single line of therapy, as recent trials in advanced KCC have done, toxic effects increase and subsequent treatment options are reduced.^13,14,15^ Thus, the relevant question facing the clinician is whether the combination of therapies is superior to the same therapies in sequence, particularly in terms of OS. Testing this hypothesis requires appropriate administration of postprotocol therapy in the patients in the control arm.^16^

We report on recent trials in first-line advanced KCC comparing combination immunotherapy regimens with the current standard of care TKI, sunitinib, and the proportion of patients in the control arm that receive postprotocol immunotherapy.

## Methods

### Overview

This study followed the relevant sections of Preferred Reporting Items for Systematic Reviews and Meta-analyses (PRISMA) reporting guideline. We sought to determine the proportion of patients who received postprotocol immunotherapy treatment in the control arms of studies comparing combination immunotherapy with sunitinib in first-line advanced KCC. We searched PubMed for clinical trials for treatment of first-line advanced KCC comparing a combination immunotherapy regimen, using 1 immunotherapy agent and either an additional immunotherapy agent or a TKI, to sunitinib in the first-line setting that were published between January 1, 2015, and February 28, 2021. We then analyzed the published articles, protocols, and supplementary materials to determine the total number of patients in the control arm, the number of patients in the control arm undergoing any form of postprotocol therapy, and the number of patients in the control arm receiving postprotocol immunotherapy, specifically, and report these figures as percentages. When available, updated results from subsequently published analyses were used.

### Data Set

#### Study Selection

We performed a PubMed search using the terms *renal cell cancer* and *first-line*, and applied the filter *clinical trial*. Trials published between January 1, 2015, and February 28, 2021, were considered. Abstracts were manually reviewed to find phase 3 trials with an intervention group of combination immunotherapy regimens and a control group of sunitinib as well as a primary outcome of PFS or OS. We excluded trials that were phase 1 or 2, subgroup analyses of other trials, not in the first-line setting, and studies other than clinical trials (cost-effectiveness, biomarker expression analysis, epidemiologic, and imaging-based studies). Each abstract was manually reviewed for inclusion or exclusion by one of us (J.S.).

#### Data Extracted

For each trial, we cataloged the National Clinical Trials number, the journal, year of publication, the countries of enrollment, number of patients enrolled by geographic region, the dates of enrollment, the treatments and doses of the intervention and control arms, the number of patients randomized to each treatment arm, the primary outcome of the trial, PFS and OS data, treatment status of patients at last data cutoff (ie, still receiving treatment or received postprotocol therapy), the number of patients in the control arm receiving postprotocol immunotherapy, and the number of patients in the control arm receiving any postprotocol therapy.

#### Status of Patients in the Control Arm at Data Close

Trial articles and supplementary materials were reviewed and CONSORT diagrams and tables summarizing postprotocol treatment were analyzed for each trial. Patients were categorized as (1) still receiving control arm therapy, (2) having received postprotocol anticancer therapy, or (3) having received no postprotocol anticancer therapy (including deceased). The patients who received postprotocol anticancer therapy were further categorized as receiving immunotherapy or receiving other anticancer therapy.

#### Rate of Postprotocol Immunotherapy Administration in Control Groups

The total number of patients in the control arm who received postprotocol immunotherapy was divided by the total number of patients who discontinued control arm treatment to provide an estimate of the proportion of patients in the control arm receiving postprotocol immunotherapy. In addition, we divided the total number of patients in the control arm who received postprotocol immunotherapy by the total number of patients in the control arm who received any form of postprotocol anticancer therapy in each trial. We then performed aggregate calculations using the same process but including all patients in all control arms across all trials considered.

#### Countries of Enrollment and Proportion of Patients

Trial figures and tables and supplementary materials were reviewed for total number of patients enrolled in each country participating in trial enrollment. Because detailed breakdowns of enrollment by country were not available in most supplements, the number of patients enrolled in each geographic region was recorded. Owing to slight heterogeneity in method of reporting across trials, we recorded patients as being enrolled in either United States/Canada/Western Europe or rest of the world.

### Statistical Analysis

We sought to provide a descriptive estimate of the proportion of patients in the control arm who received postprotocol immunotherapy after first-line treatment with sunitinib for advanced KCC. Analysis was performed using Microsoft Excel (Microsoft Corp).

## Results

**Figure 1.**
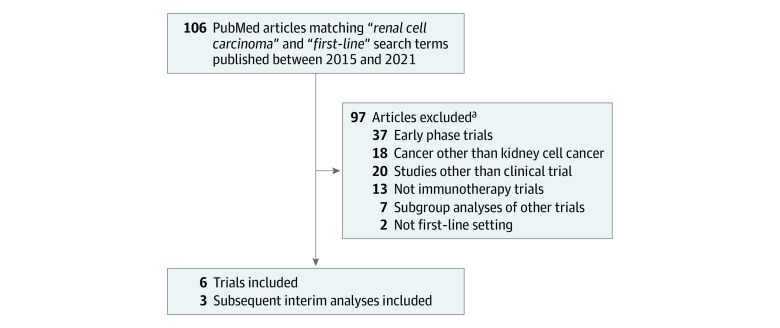
Flow Diagram of Systematic Review Selection Criteria ^a^Many articles met multiple inclusion criteria but were counted once for the predominant exclusion criterion they met.

A total of 106 studies met our search criteria. The reasons studies were excluded included that they involved phase 1 or 2 trials (37), cancer other than KCC (18), subgroup analysis of other trial (7), no immunotherapy component (13), not first-line setting (2), and study type other than clinical trial (20). Several studies met more than one exclusion criteria. We identified 6 clinical trials and 3 updated analyses comparing immunotherapy combination regimens with sunitinib in first-line advanced KCC that were published between January 1, 2015, and February 28, 2021. One trial, CLEAR, had a second intervention arm of lenvatinib and everolimus but because this regimen did not include an immunotherapy agent, this group of patients was not included in our analysis. All trials used identical control arm treatments of sunitinib (50 mg once a day with identical dose reduction schemes). Three trials, KEYNOTE-426,^11^ CheckMate 214,^17^ and JAVELIN Renal 101,^9^ had subsequently published analyses that were used. Figure 1 summarizes our search strategy. The Table summarizes the studies we considered in our analysis.^8,9,10,11,12,17^

**Table.** Postprotocol Immunotherapy Administration Characteristics in Trials of Combination Immunotherapy Regimens vs TKI for First-line Advanced Kidney Cell Carcinoma

Trial	Intervention arm	Control arm	Total patients assigned to control arm	Patients in the control arm, No. (%)				
				Discontinued TKI	Receiving any postprotocol therapy	Receiving postprotocol immunotherapy	Receiving immunotherapy as percentage of those who discontinued TKI	Receiving immunotherapy as percentage of those who received any postprotocol therapy
CLEAR (NCT02811861)^10^^,^^a^	Pembrolizumab + lenvatinib	Sunitinib	357	290 (81.2)	206 (57.7)	154 (43.1)	154/290 (53.1)	154/206 (74.8)
CheckMate 9ER (NCT03141177)^12^	Nivolumab + cabozantinib	Sunitinib	328	236 (72.0)	108 (32.9)	67 (20.4)	67/236 (28.4)	67/108 (62.0)
IMmotion151 (NCT02420821)^8^	Atezolizumab + bevacizumab	Sunitinib	461	323 (70.1)	238 (51.6)	144 (31.2)	144/323 (44.6)	144/238 (60.5)
KEYNOTE-426 (NCT02853331)^11^^,^^b^	Pembrolizumab + axitinib	Sunitinib	429	353 (82.3)	242 (56.4)	169 (39.4)	169/353 (47.9)	169/242 (69.8)
JAVELIN Renal 101 (NCT02684006)^9^^,^^b^	Avelumab + axitinib	Sunitinib	444	336 (75.7)	227 (51.1)	159 (35.8)	159/336 (47.3)	159/227 (70.0)
CheckMate 214 (NCT02231749)^17^^,^^b^	Nivolumab + ipilimumab	Sunitinib	546	531 (97.3)	382 (56.4)	239 (43.8)	239/531 (45.0)	239/382 (62.6)
Total	Combination immunotherapy	Sunitinib	2565	2069 (80.7)	1403 (54.7)	932 (36.3)	932/2069 (45.0)	932/1403 (66.4)

Abbreviation: TKI, tyrosine kinase inhibitor.

^a^ An additional intervention arm of lenvatinib or everolimus was not included in our analysis because it did not include an immunotherapy component.

^b^ Published updates of results for these trials were used for calculations.

### Treatment Status of Patients in the Control Arm at Last Data Cutoff

**Figure 2.**
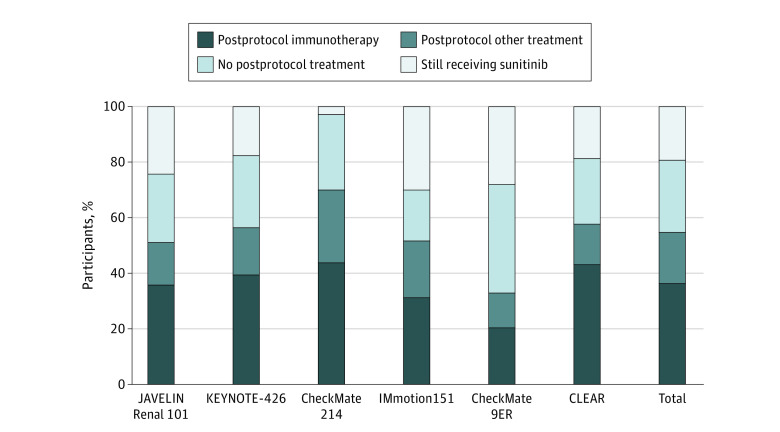
Treatment Status of Patients Randomized to Control Arm Treatment With Sunitinib at Time of Last Data Cutoff by Trial and in Total

In total, 2565 patients were assigned to control arms across all trials. At last data cutoff, 496 patients (19.3%; range, 2.7%-29.9%) across all trials continued to receive sunitinib treatment. A total of 666 patients (26.0%; range, 18.4%–39.0%) who discontinued sunitinib received no postprotocol therapy. A total of 1403 patients (54.7%; range, 32.9%–70.0%) who discontinued sunitinib received some form of postprotocol therapy. A total of 932 patients (36.3%; range, 20.4%-43.8%) received postprotocol immunotherapy and 471 patients (18.4%; range, 12.5%-26.2%) received some other form of postprotocol therapy. Figure 2 summarizes the treatment status of patients in the control arm at last data cutoff in each trial. As a proportion of patients who discontinued sunitinib, 45.0% (range, 28.4%-53.1%) received postprotocol immunotherapy. As a proportion of patients receiving any form of postprotocol therapy, 66.4% (range, 60.5%-74.8%) received immunotherapy. The Table summarizes the proportion of patients receiving postprotocol immunotherapy by trial.

### Multinational Trials

All 6 trials were conducted in the multinational setting. In total, 4872 patients were enrolled (our analysis does not consider the 357 patients randomized to lenvatinib or everolimus in CLEAR). Of these patients, 2759 (56.6%) were enrolled in the US, Canada, or Western Europe. A total of 2113 (43.4%) were enrolled in the rest of the world, which included Argentina, Australia, Brazil, Chile, Colombia, Israel, Mexico, New Zealand, Russia, South Korea, Taiwan, and Ukraine.

## Discussion

In trials comparing immunotherapy combination regimens with sunitinib in first-line advanced KCC, we found that 45.0% of patients who discontinued control arm treatment received postprotocol immunotherapy. We additionally found that, of patients in the control arm who received postprotocol therapy of any kind, only 66.4% received immunotherapy.

To our knowledge, this is the first investigation of control arm postprotocol immunotherapy administration after first-line treatment in advanced KCC. This is a notable area of inquiry because there are limited proven effective therapies for this disease, and appropriate use of lines of therapy is essential to provide the best outcomes for patients. While providing combination immunotherapy regimens in the first-line setting has been reported to improve PFS in each of these trials, a relevant clinical question is whether the strategy of using multiple agents in a single treatment line, and thus depleting subsequent treatment options, is associated with improved OS compared with sequential therapy.

While median OS had not been reached at the time of publication in any of the 6 trials we considered,^8,9^ 4 of the trials report that combination immunotherapy was associated with a statistically significant improvement in OS.^10,11,12,17^ In published subsequent analyses,^18,19,20^ OS hazard ratios continued to favor combination immunotherapy treatment in KEYNOTE-426 and CheckMate 214, and median OS remained unreached in these treatment arms. The low use of postprotocol immunotherapy raises the question that some of the observed OS benefit may be owing to suboptimal postprotocol therapy in the control arm instead of better efficacy with up-front combination therapy.

The sites of enrollment may help to explain the low rate of postprotocol immunotherapy in patients in the control arm. These therapies are expensive and not always widely available in every country. Oncologists using these trials to make treatment decisions must be aware of how readily available immunotherapy is where they practice and consider whether the rates of administration reported in these trials reflect their experience in the clinic. Discrepancies in the rate of post–TKI immunotherapy between these trials and real-world practice should be acknowledged when interpreting the reported OS data and making treatment decisions.

### Limitations

This study has limitations. First, the 2 figures we report of patients in the control arm receiving postprotocol immunotherapy (percentage by number that discontinued control arm treatment, percentage by number receiving any postprotocol therapy) are both surrogates for the truly meaningful figure: percentage by number fit to receive postprotocol immunotherapy. We acknowledge that, for some patients who did not receive postprotocol immunotherapy but did receive other postprotocol therapy, this decision was likely appropriate owing to comorbidities, decreases in global health status, or other reasons. Adjusting for these patients, the percentage of patients who appropriately receive postprotocol immunotherapy is likely a slightly higher figure than the one we report of 66.4% of patients receiving any form of postprotocol therapy. However, it is unlikely that the entirety of the remaining 33.6% of patients would have been considered unfit for postprotocol immunotherapy. Furthermore, it is possible that at least a portion of the 26.0% of patients who discontinued sunitinib but received no postprotocol therapy may have been fit for immunotherapy.

Second, the studies we considered have different follow-up periods and therefore have different numbers of patients who progressed receiving control arm therapy and were eligible for postprotocol therapy. We performed our calculations based on patients who have discontinued control arm treatment instead of the intention-to-treat population so that studies with shorter follow-up periods would not be characterized as having low rates of postprotocol immunotherapy administration owing to a larger proportion of patients receiving control arm treatment. By reporting a complete breakdown of the treatment status of all patients in the control arm at last data cutoff in Figure 2, including the number of patients who continued to receive control arm treatment with sunitinib and the number who have received other postprotocol treatment as well as those receiving no subsequent treatment after study discontinuation, we have provided data to support the finding of a low rate of postprotocol immunotherapy administration that is not fully explained by a shorter follow-up period.

Third, we recognize that the binary geographic groupings of US/Canada/Western Europe and rest of the world lacks sufficient specificity to provide a more nuanced discussion regarding local availability of postprotocol immunotherapy. We report these data in this manner because more granular country-by-country data on patient enrollment were not uniformly available in the articles or supplements. We believe the multinational nature of these trials likely affects the availability of postprotocol immunotherapy and have attempted to quantify how much this may have affected the results we observed, but without more detailed data being uniformly available, we acknowledge that future studies are needed to validate this hypothesis.

## Conclusions

Advanced KCC remains a challenging disease to treat with a limited number of effective therapies. While recent trials comparing combination immunotherapy regimens with the TKI sunitinib in the first-line setting have reported positive, if preliminary, OS results, interpretation of these figures must be informed by the rate of appropriate postprotocol immunotherapy in patients in the control arm. We found that the proportion of patients in the control arm receiving postprotocol immunotherapy, which has a proven mortality benefit, is low. This raises the question of how much of the benefit of first-line combination immunotherapy regimens is associated with superior efficacy of the combination regimen compared with a lack of postprotocol immunotherapy treatment in the control arm.
